# Characterization of the complete chloroplast genome of a medicinal plant, *Portulaca oleracea*

**DOI:** 10.1080/23802359.2020.1791016

**Published:** 2020-07-23

**Authors:** Chen Li, Jinsong Li, Tianyi Cao, Kun Li, Tao Tang

**Affiliations:** a Xiangya Stomalogical Hospital & School of Stomatology, Changsha, P. R. China; b Jining Second People’s Hospital, Jining, P. R. China; c Department of Hand Surgery, Zhejiang Province People’s Hospital Haining Hospital, Haining, P. R. China; d Department of Oral and Maxillofacial Surgery, Xiangya Stomalogical Hospital & School of Stomatology, Changsha, P. R. China; e Institute of Integrative Medicine, Xiangya Hospital, Changsha, P. R. China

**Keywords:** *Portulaca oleracea*, chloroplast genome, phylogenetic relationship, phylogenetic analysis

## Abstract

*Portulaca oleracea* is an important and widely distributed medicinal and edible plant, which has great economic value in the medical and food industries in the whole world. The complete chloroplast genome of *Portulaca oleracea* was found to possess a total length 156,533 bp and had the circular structure. It was the typical quadripartite structure the same as many plants, including 87,437 bp large single-copy region (LSC), 18,096 bp small single-copy region (SSC) of and a pair of 25,500 bp inverted repeat (IR) regions. The total of 132 genes was successfully annotated, which contained 87 protein-coding genes, 37 transfer *RNA* genes, and eight ribosomal *RNA* genes. Nineteen genes were found in one of IR, which included eight protein-coding gene, seven *tRNA* genes, and four *rRNA* genes. The overall nucleotide composition of chloroplast genome sequence is: 31.5% (A), 32.1% (T), 18.5% (C), 17.9% (G), and the total GC content of 36.4%. The phylogenetic analysis result showed that *Portulaca oleracea* was close to *Carnegiea gigantean in* the phylogenetic relationship by the neighbor-joining (NJ) method in this study.

*Portulaca oleracea* is listed by the World Health Organization (WHO) as one of the most used medicinal and edible plants, which is packed full of nutrients, vitamins, and minerals. *Portulaca oleracea* has been widely used as a potherb and herb in the Mediterranean, central European, and Asian countries (Iranshahy et al. 2017). *Portulaca oleracea* has been used as a traditional Chinese medicine (TCM) in China, acting as a febrifuge, antiseptic, vermifuge, and so forth (Rahimi et al. 2019). It is considered as a beneficial herb in the world and contains antimicrobial, antidiabetic, and anti-inflammatory properties (Iranshahy et al. 2017). In this study, the complete chloroplast genome of *Portulaca oleracea* was characterized and generated, which can be used for the active ingredient of TCM and drug development in this species.

The fresh *Portulaca oleracea* were collected from the Hunan University of Chinese Medicine that located at Changsha, Hunan, and China (112.90E, 28.13 N), which were stored in the −80 °C refrigerator. Total genomic DNA of *Portulaca oleracea* was extracted from the fresh leaves with a modified CTAB method and sequenced, which was stored in Hunan University of Chinese Medicine (No.HNUCM-001). Here, the quality controlled and removed the sequences used by FastQC (Andrews 2015). The chloroplast genome of *Portulaca oleracea* was assembled by MitoZ (Meng et al. 2019) and annotated by geneious (Kearse et al. 2012). All the coding and other genes were predicted by CPGAVAS (Liu et al. 2012) and NCBI Blast (https://blast.ncbi.nlm.nih.gov/Blast.cgi) in the chloroplast genome. Finally, the circular chloroplast genome map was generated by DOGMA (Wyman et al. 2004).

The complete chloroplast genome of *Portulaca oleracea* (NCBI No. NK9714231) was found to possess a total length 156,533 bp and had the circular structure. It was the typical quadripartite structure the same as many plants, including 87,437 bp large single-copy region (LSC), 18,096 bp small single-copy region (SSC) of and a pair of 25,500 bp inverted repeat (IR) regions. The total of 132 genes was successfully annotated, which contained 87 protein-coding genes, 37 transfer *RNA* genes, and 8 ribosomal *RNA* genes. Here, 19 genes were found in one of the IR, which included eight protein-coding genes (*rps19*, *rpl2*, *rpl23*, *ycf2*, *ndhB*, *rps7*, *rps12*, and *ycf1*), seven *tRNA* genes (*trnI-CAU*, *trnL-CAA*, *trnV-GAC*, *trnI-GAU*, *trnA-UGC*, *trnR-ACG*, and *trnN-GUU*) and four *rRNA* genes (*rrn16*, *rrn23*, *rrn4.5*, and *rrn5*). The overall nucleotide composition of chloroplast genome sequence is: 31.5% (A), 32.1% (T), 18.5% (C), 17.9% (G), and the total GC content of 36.4%, respectively.

The neighbor-joining (NJ) tree was reconstructed using the complete chloroplast genome sequence of *Portulaca oleracea* with other published 12 species from NCBI (Figure 1). All the sequences from NCBI were aligned by MAFFT (Katoh and Standley 2013) and the NJ phylogenetic analyses were conducted by MEGA X (Kumar et al. 2018). We used MEGA X with 2000 bootstraps values under the substitution model to reconstruct the NJ phylogenetic tree. At last, the NJ phylogenetic tree was drawn and edited using MEGA X (Kumar et al. 2018). The phylogenetic analysis result showed that *Portulaca oleracea* was close to *Carnegiea gigantean in* the phylogenetic relationship by the NJ method in this study. The result can be used for the active ingredient of TCM and drug development in this species.

**Figure 1.**
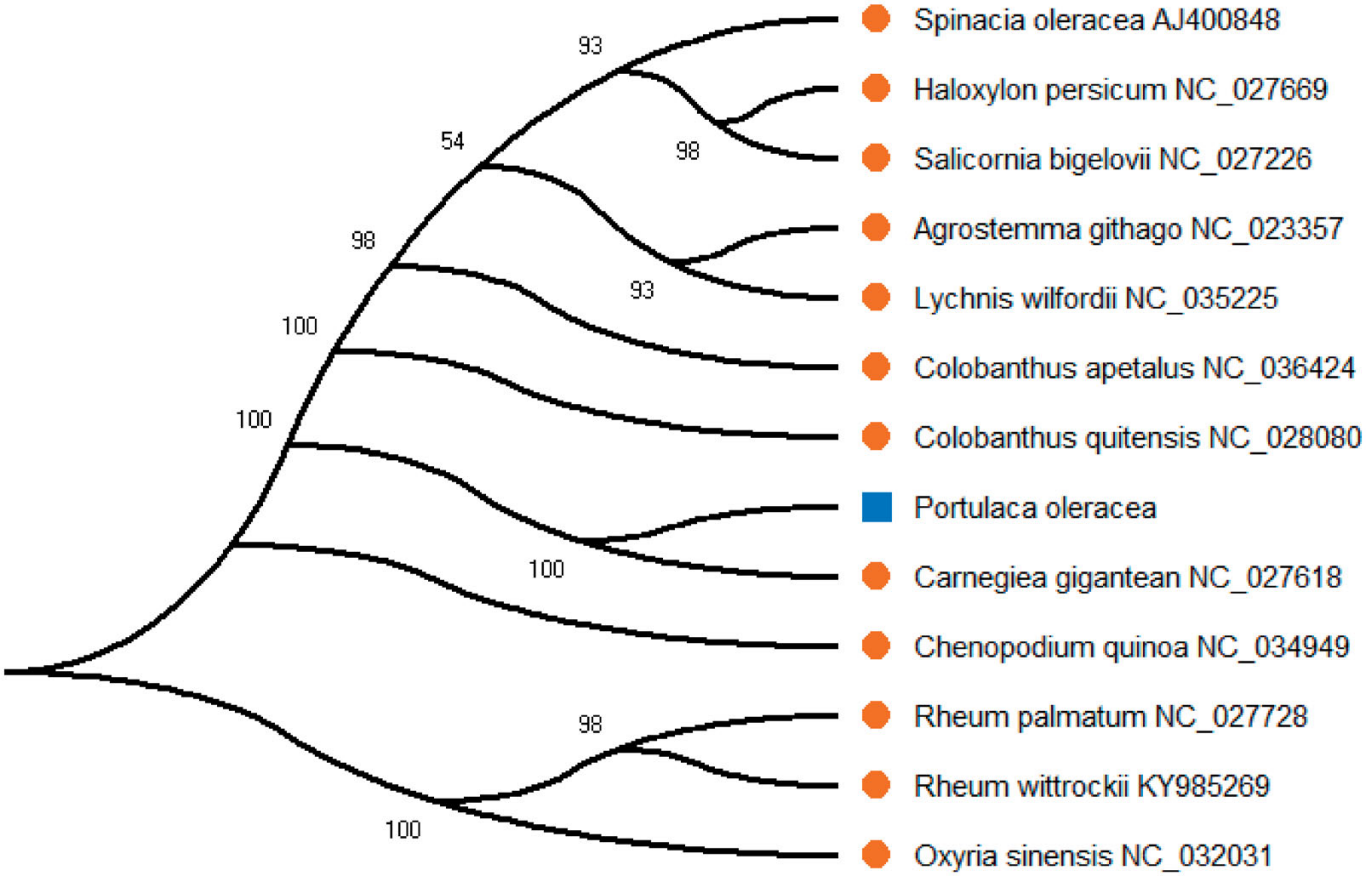
The neighbor-joining (NJ) phylogenetic tree based on 13 plant species complete chloroplast genome sequences.

## Data Availability

The data that support the findings of this study are openly available in *Portulaca oleracea* at http://doi.org/10.1080/23802359.2020.1791016, reference number [reference number NK9714231]. The data that support the findings of this study are available from the corresponding author, upon reasonable request.
